# Efficacy, Feasibility, and Safety of a Game-Based Home Exercise Program to Improve Balance: A Pilot Study

**DOI:** 10.1093/geroni/igaf122.3755

**Published:** 2025-12-31

**Authors:** Jordan Kurth, Emily Cornish, Alexander Wiggins, Allisyn Hetherington, Robert Creath, Christopher Sciamanna

**Affiliations:** Penn State College of Medicine, Hershey, Pennsylvania, United States; Penn State College of Medicine, Hershey, Pennsylvania, United States; Lebanon Valley College, Annville, Pennsylvania, United States; Penn State College of Medicine Milton S. Hershey Medical Ctr, Hershey, Pennsylvania, United States; Lebanon Valley College, Annville, Pennsylvania, United States; Penn State College of Medicine, Hershey, Pennsylvania, United States

## Abstract

One-third of older adults fall each year, leading to loss of independence and costing billions. While exercise is widely proven to reduce falls, many older adults do not do enough to benefit. Game-based activities are inherently more enjoyable than longer, traditional exercise, but no study has examined the impact of brief, game-based workouts on balance. Our objective was to understand the preliminary efficacy, feasibility, and safety of a 5-minute daily game-based exercise program among older adults at-risk of falling. A total of 16 participants (12 female; mean age 72 years) who had fallen in the past year and performed less than 60 minutes of weekly moderate-to-vigorous exercise completed the 2-week intervention. The intervention involved 3x1-minute sessions (followed by 1-minute rests) of paddling a foam ball against a wall in their home, trying to prevent the ball from hitting the ground. Study staff met virtually with each participant once over the two-week intervention. Timed Up-and-Go scores decreased by an average of 0.76 seconds from baseline (9.35s) to follow-up (8.59s; SMD = -0.5). Average 6-minute walk test distances improved by an average of 30 meters from baseline (341m) to follow-up (371m; SMD 0.48). Zero participants reported a fall. Adherence was also extremely high (98% of days). The average program satisfaction score was 8.5/10. Brief, game-based at-home exercise programs appear to be effective, feasible, and safe for older adults at risk of falling. Future randomized controlled trials should be conducted to test program efficacy on clinically-relevant outcomes over a longer period of time.

